# Seasonal distribution and upsurge of respiratory viruses among indigenous tribes with ILI and SARI in a far-flung Car Nicobar Island

**DOI:** 10.1186/s12879-024-09536-1

**Published:** 2024-06-28

**Authors:** Nagarajan Muruganandam, Veena Vipat, Sheetal Jadhav, Alwin Vins, Nisha Beniwal, Harpreet Kaur, Remya Rajan Renuka, Rehnuma Parvez, Varsha Potdar

**Affiliations:** 1 Indian Council of Medical Research – Regional Medical Research Centre, Port Blair City, Andaman and Nicobar Islands India; 2 Indian Council of Medical Research – National Institute of Virology, Pune City, Maharashtra India; 3 Indian Council of Medical Research – Headquarters, Ansari Nagar, New Delhi, India; 4 Centre for Global Health Research, Saveetha Institute of Medical and Technical Sciences, Chennai City, Tamil Nadu India

**Keywords:** Influenza, RSV, Island, Respiratory virus, ILI, Nicobarese

## Abstract

**Background:**

Respiratory viral illnesses among children are a prominent cause of morbidity and mortality in the developing world. The aim of this study is to understand the seasonal pattern and surge of respiratory viruses among the Nicobarese tribe.

**Methods:**

Respiratory specimens were collected from both ARI and SARI cases attended the BJR district hospital in Car Nicobar Island, India, between 2021 and 2022. Respiratory viruses were identified from the specimens by using the qRT-PCR assay. Meteorological parameters were collected and evaluated using Microsoft Excel and SPSS 21. The significant association between the surge of respiratory viruses and each climatic parameter was evaluated.

**Results:**

In this hospital-based cross-sectional study, 471 ILI cases were enrolled, and 209 of these were positive for respiratory viral infections. Of these respiratory virus infections, 201 (96.2%) were infected with a single respiratory virus infection, and 8 (3.8%) had mixed viral infections. Fever, cough, and chills were the most common symptoms of respiratory illness among this indigenous population. There was a significant link between respiratory viruses and influenza-like illness in children (below 5 years and 6 to 15 years).

**Conclusion:**

This prevalence study revealed that viral respiratory infections were more common in children than adults. Among these respiratory viruses, respiratory syncytial virus A (RSV) and influenza B virus were predominantly reported among tribal children up to age five years. In the year 2021, these viruses were recorded frequently during the winter season. Climate factors such as high humidity, high precipitation, moderate temperature, and moderate rainfall are found to be correlated with respiratory viral infections. This study implicates important information for preventing a further outbreak of respiratory viral infections in Car Nicobar Island.

**Supplementary Information:**

The online version contains supplementary material available at 10.1186/s12879-024-09536-1.

## Introduction

Human respiratory virus infections cause a wide array of respiratory symptoms and severe illness, resulting in morbidity, mortality, and economic losses worldwide. According to the Global Burden of Disease (GBD) 2019 report, the global burden of respiratory infections reached 17.2 billion in 2019 [1]**.** Fever, cough, sore throat, nasal stiffness, and headache are the most frequent symptoms of acute respiratory infections (ARIs) [2]. A significant range of respiratory viruses that infect humans have been discovered to be extremely contagious for individuals of various ages [3]. In addition to influenza viruses, respiratory syncytial virus (RSV), human meta-pneumovirus (hMPV), human coronaviruses (HCoV), adenoviruses (AdV), and parainfluenza viruses (PIV) have been associated to infections in both children and adults [4, 5]**.**

Climatic factors are associated with an increase in the circulation of respiratory viruses at particular duration of a year. Furthermore some respiratory viruses like RSV and Influenza virus were appeared during winter season. However, Rhinovirus had two upsurge that occurs during October to November and in March. Moreover, Adenovirus was circulated throughout the year. RSV and Influenza A virus were prevailing in the high relative humidity and low temperature. Nevertheless, HMPV will circulate in the warmer temperature [6].

The Nicobar Islands in the Indian Ocean are located approximately 1,300 km southeast of the Indian subcontinent, across the Bay of Bengal (1,841 square km). The Nicobar Islands, along with the Andaman Islands to the north, constitute the boundary between the south-eastern Bay of Bengal (west) and the Andaman Sea (east) [7]**.** Car Nicobar is a small (49 square kilometers) remote island that can be reached by sea or air from Port Blair, the main city of the Andaman and Nicobar Islands. It is situated 260 km from Port Blair. The Nicobarese tribes (mongoloids), one of six indigenous aboriginal tribes of Car Nicobar Island, have a population of 17,841 (> 98% Nicobarese) as of the 2011 census, and has a tropical climate since it lies 9 degrees south from the equator [8]. The climate in the Andaman and Nicobar Islands is varied, with both the first and second monsoons bringing heavy rain. The regular travel of this tribe to Port Blair for personal chores, where tourists visit from India and all over the globe especially, raises the possibility of many respiratory infections spreading to this remote island.

Children's respiratory disorders are a growing concern for morbidity and mortality in developing countries, especially, when they are linked to chronic respiratory diseases [9]**.** Due to weakened immunity and other factors, respiratory infections were more common in children and adults with influenza-like illness (ILI) and severe acute respiratory infection (SARI) [10]**.**

Our previous study from Car Nicobar Island revealed the existence of several respiratory viral infections among Nicobarese tribes, with influenza virus being the most prevalent [11]. A deeper understanding of seasonal patterns, if any, in relation to respiratory virus infections among the indigenous community in the Car Nicobar Island is required. Therefore, the aim and objective of this study was to identify respiratory viruses and comprehend the climatic factors related to these viruses infections among the Nicobarese tribe in India during the years 2021 and 2022.

## Methodology

### Study design and population

A hospital-based cross-sectional study was carried out at the BJR hospital in Car Nicobar Island, Andaman and Nicobar Islands, India (1st June 2021 to 31st May 2022). A total of 5840 patients of various age groups from the aboriginal Nicobarese tribe reported to the BJR hospital throughout the study period.

### Study area

Car Nicobar (9.1573° N, 92.7581° E) is a small island that lies in proximity to neighboring countries, i.e. Myanmar, Malaysia, and Indonesia. The Nicobarese tribe has inhabited this Island, which is located near the equator and has a tropical climate.

### Case definition

#### Influenza-like illness (ILI)

An acute respiratory infection with measured fever of ≥ 38 °C and a cough with onset within the 10 days [12].

#### Severe acute respiratory illness (SARI)

Acute respiratory illness requiring hospitalization that has started within the past 10 days and has a history of fever or a recorded fever of ≥ 38 °C as well as cough [12].

#### Inclusion criteria

Patients having symptoms of acute respiratory tract infections, primarily ILI and SARI were included.

#### Exclusion criteria

Patients with confirmed TB, chronic respiratory illness such as lung cancer, COPD and Asthma, patient with bacterial infections and patients unwilling to participate in the study were excluded from the study.

### Collection of samples

The collection of respiratory specimens including throat and nasal swabs was done for the patients who visited BJR Hospital during the study period with suspected cases of ILI and SARI after obtaining written informed consent from the patients. In the case of children, the written consent of the parent or legal guardian was obtained. The respiratory sample was taken with the assistance of a physician or a skilled nurse. Furthermore, the collected samples were handled and transported according to the standard operating procedures [13].

### Data collection

The participants in this study were recruited based on their presenting signs and symptoms between June 2021 and May 2022. Data related to the socio-demographic profile, travel history, and clinical manifestations were collected through the use of a pre-established questionnaire (Supplementary file 1).

The personal information, clinical observations, and epidemiological data pertaining to these cases were documented in the clinical proforma. Data was obtained from patients diagnosed with Influenza-like Illness (ILI) and Severe Acute Respiratory Infection (SARI) in various hospital settings at BJR Hospital in Car Nicobar, India. The data was entered into Microsoft Excel version 2016. The ILI and SARI cases were chosen in accordance with specific inclusion criteria, symptoms, and case definition.

### Laboratory testing

Ribonucleic acid (RNA) was extracted using the MagMax-96 viral RNA isolation kit (Invitrogen, Thermo-Fischer Scientific, USA) following the manufacturer's instructions. Utilizing the Invitrogen Superscript III one-step quantitative RT-PCR kit, real-time reverse transcription PCR (qRT-PCR) was performed using a set of previously published primers and probes to detect respiratory viruses including Human Coronavirus-229E (HCOV -229E), Organ Culture 43 (OC43), Hong Kong University 1 (HKU1), NetherLand 63 (NL63), influenza A virus, influenza B virus, human adenovirus (HAdV), respiratory syncytial virus (RSV) A and B, human metapneumovirus (hMPV), para-influenza virus (PIV) 1, 2, 3, and 4, and rhinovirus (Invitrogen, Thermo Fischer Scientific, USA) [14].

Type-specific primers and probes for HCoV 229E, NL63, and OC43 were chosen based on the N gene's highly conserved genomic regions. The fluorogenic probes of HCoV 229E, NL63, and OC43 that can be labelled with several fluorogenic dyes were labelled with a 5′ reporter dye, 6-carboxy-fluorescein, and a 3′ quencher dye, 6-carboxytetramethyl-rhodamine [14]. The ABI 7500 equipment was used for this RT-PCR test (Applied Biosystems Inc, USA). A total of 25 µL of PCR reaction mixture was prepared using 10 µmol of forward and reverse primers, 5 µmol of Taqman probe, 12.5 µL of 2X buffer, 0.5 µL of superscript TM III enzyme, and 5 µL of nucleic acid template. The PCR Assay took 45 cycles to complete, including an initial denaturation at 94 °C for 5 min, denaturation at 94 °C for 15 s, and annealing at 55 °C for 30 s [11, 14]. The identification of all target viruses was confirmed using a positive and negative controls consisting of in vitro transcribed RNAs.

### Meteorological data

Car Nicobar Island encompasses (9.17°N 92.78°E) is extremely level, with the exception of few cliffs in the north and a few mountainous places in the interior. Daily data of Rainfall (in millimeter) and relative humidity (in percentage) for Car Nicobar Island from June 01, 2021 to May 31, 2022 were gathered from IMD, Pune's CEIC data (https://www.ceicdata.com/en/). Moreover, NOAA (National Oceanic and Atmospheric Administration), Deutscher Wetterdienst, Canada (https://meteostat.net/en/) provided the daily data of temperature (in degree Celsius) and precipitation (in millimeter). Monthly average (mean) data (June, 2021 to May, 2022) of rainfall, relative humidity, temperature, and precipitation for Car Nicobar Island were analyzed using Microsoft Excel. Month wise mean data was compared with the monthly distribution of respiratory viruses during the same period.

### Statistical data analysis

Surveillance data of respiratory viruses among Nicobarese in Car Nicobar were entered, and stored in Microsoft Excel version 2016, and. Descriptive statistics of was performed by using SPSS 21(SPSS Inc., Chicago, IL, USA). Chi-square test was used to compare the categorical data. In addition, month wise data of climatic factors (rainfall, relative humidity, temperature, and precipitation) were compared with various respiratory viruses reported during the same period using a T-test. *P*-value less than 0.05 were considered as significant.

### Ethical consideration

The institutional ethical committee of the ICMR—Regional Medical Research Centre, Port Blair, approved the study (Proposal No.9) on January 11, 2019. Respiratory specimens were obtained in accordance with human ethical rules and requirements. Informed consent was obtained from each patient who participated in this study, and in the case of minors (children and adolescents), informed consent from a parent and/or legal guardian was sought.

## Result

### Demographic and clinical characteristics

In this study, 5840 patients with influenza-like illness visited to the outpatient and inpatient settings of BJR District hospital in Car-Nicobar. Respiratory samples were collected from 471 enrolled patients of the total 5840 patients with ILI, and tested for respiratory viruses. The median age of ILI and SARI patients was 22 years old (IQR: 7 – 36 years). The median age of ILI cases attending outpatient settings was high compared to the hospitalized cases (M = 9, IQR: 3–32 vs. M = 3, IQR: 1–8.5). Out of the total 5840 individuals with influenza-like illness (ILI), 212 patients (3.6%) were admitted to the hospital due to severe acute respiratory illness (SARI), while the remaining 5628 cases (96.4%) have been treated as outpatients. The majority of the patients attended the outpatient setting was compared with hospitalized cases. Compared to inpatient settings, more women were attended in outpatient settings (51.6% vs. 46.2%).

**Fig. 1:**
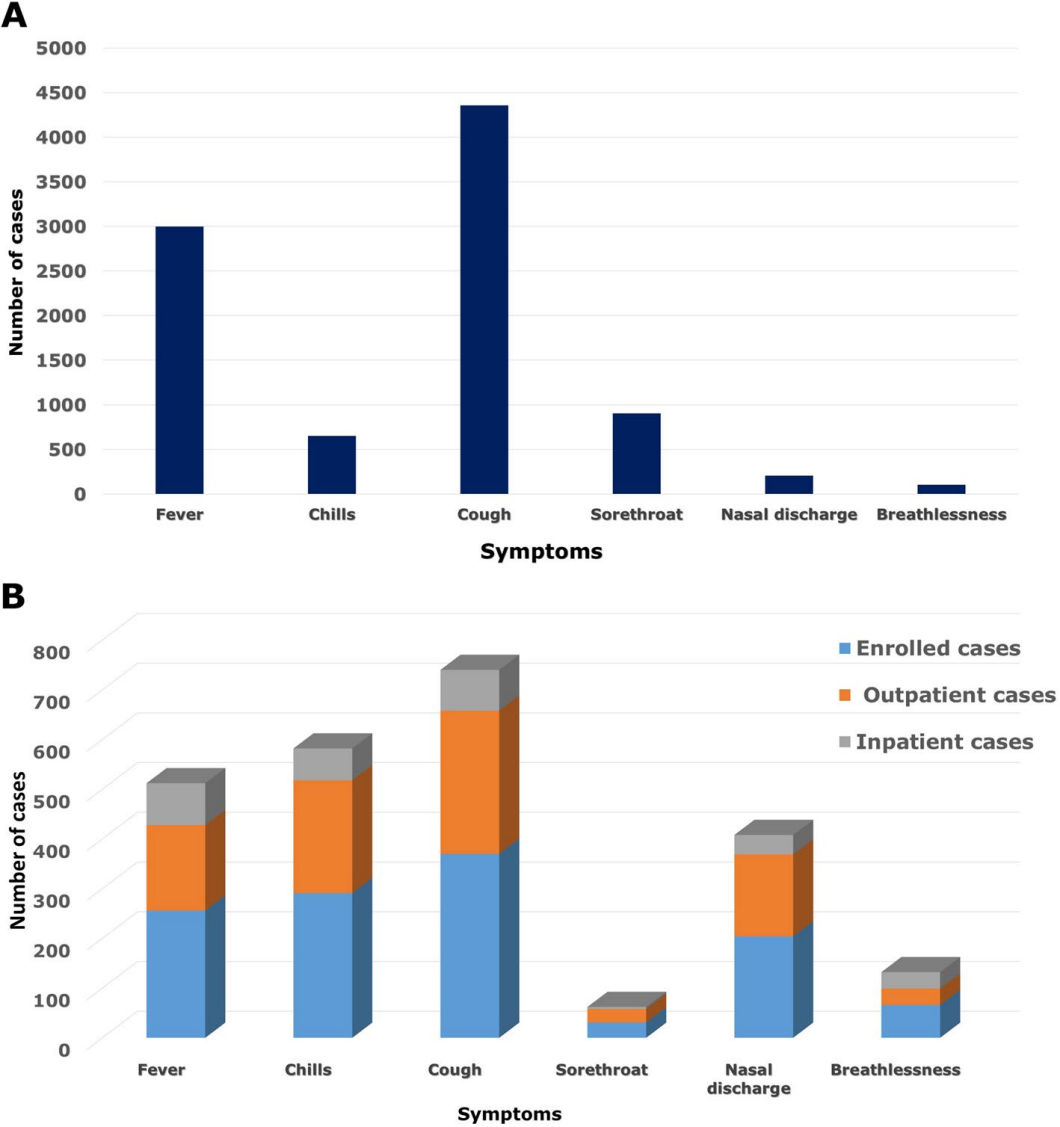
Symptomatic distribution of ILI in Car-Nicobar (**A** shows the symptomatic distribution of total ILI cases attended the hospital (*N* = 5840) & **B** depicts the symptomatic distribution of enrolled ILI cases in this study (*N* = 471)

Nevertheless, the percentage of male patients attending the outpatient settings was lower than the hospitalized cases (OPD vs. IPD: 78.5% vs. 21.5%). Hospitalized (inpatient) settings had a higher frequency of instances (67.7%) than outpatient settings among children under the age of five. The demographic features of ILI in Car-Nicobar are elucidated in Table 1. Among these 5840 ILI cases, the most common symptoms were cough (74.6%), fever (51.4%), sore throat (15.5%), chills (11.1%), and runny nose (3.5%). Figure 1 illustrates the symptomatic distribution of ILI patients in Car Nicobar.

**Table 1:** Demographic characteristics of patients with ILI in Car-Nicobar, India (2021 – 2022)

Characteristics	Total ILI Cases (N, %) (*N* = 5840)	Enrolled ILI cases (N, %) (*N* = 471)	Inpatient settings (N, %) (*N* = 93)	Outpatient settings (N, %) (*N* = 378)	Positive cases (N, %) (*N* = 209)	*P*-value
**Gender**						
Male	3193 (54.7)	233 (49.5)	50 (53.8)	183 (48.4)	101 (48.3)	*P* < 0.05
Female	2647 (45.3)	238 (50.5)	43 (46.2)	195 (51.6)	108 (51.7)	*P* < 0.05
**Median Age (in years) (IQR)**	22 (7–36)	7 (2–30)	3 (1–8.5)	9 (3–32)	3 (1–8)	*P* < 0.05
**Age** (years)						
0 – 5	1205 (20.6)	208 (44.2)	63 (67.7)	145 (38.4)	139 (66.5)	*P* < 0.05
6 _ 15	1040 (17.8)	88 (18.7)	12 (12.9)	76 (20.1)	33 (15.8)	*P* < 0.05
16 – 25	1043 (17.9)	41 (8.7)	1 (1.1)	40 (10.6)	12 (5.7)	*P* < 0.05
26 – 35	1057 (18.1)	41 (8.7)	6 (6.5)	35 (9.3)	6 (2.9)	*P* < 0.05
36 – 45	656 (11.2)	32 (6.8)	2 (2.2)	30 (7.9)	7 (3.3)	*P* < 0.05
46 – 55	458 (7.8)	28 (5.9)	3 (3.2)	25 (6.6)	7 (3.3)	*P* < 0.05
56 – 65	252 (4.3)	20 (4.2)	2 (2.2)	18 (4.8)	2 (1.0)	*P* = 0.17
≥ 66	129 (2.2)	13 (2.8)	4 (4.3)	9 (2.4)	3 (1.4)	*P* < 0.05

### Detection of respiratory viruses

**Fig. 2:**
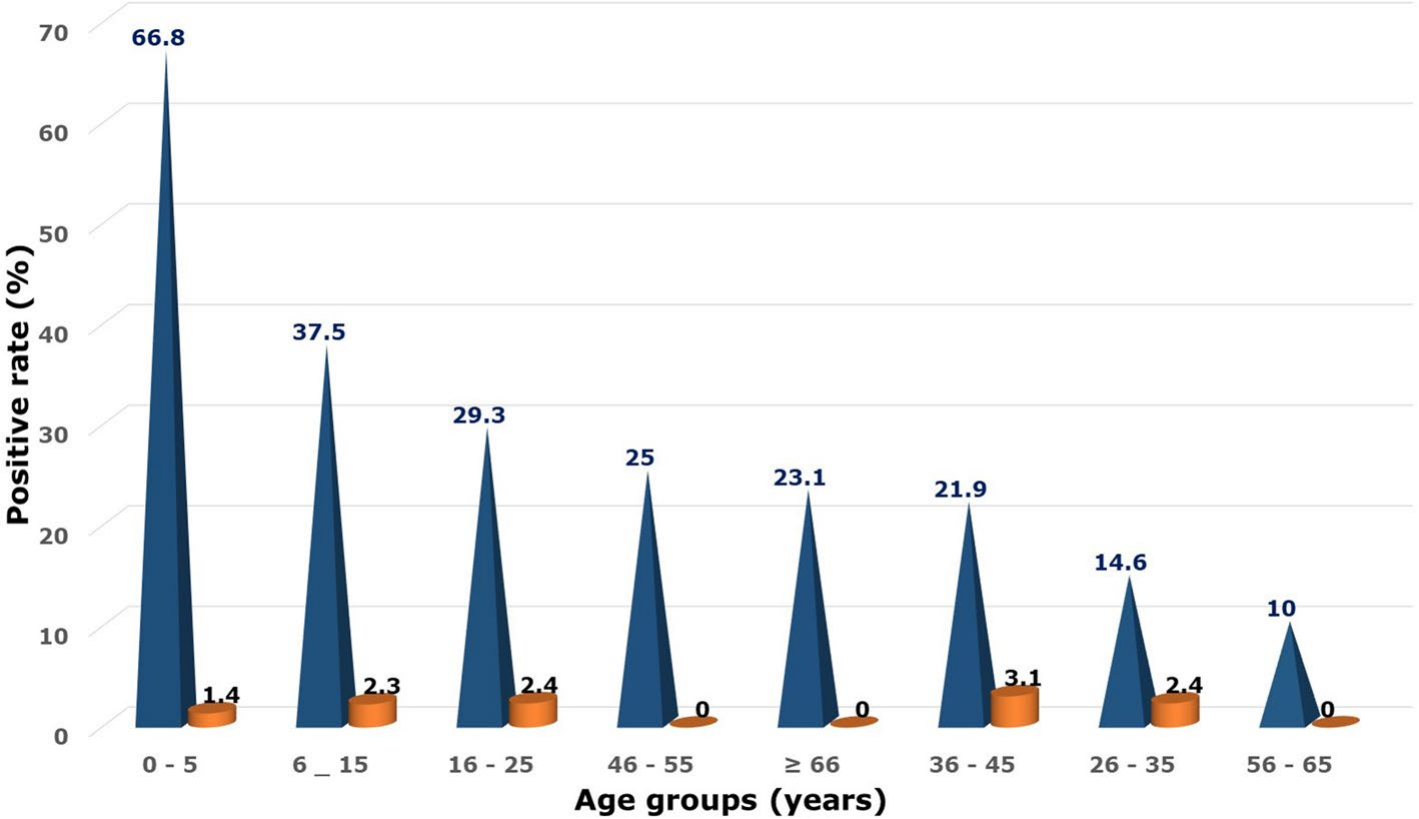
Positive rates of single viral respiratory infection and viral-viral co-infection with different age groups (Blue cone indicates the single viral respiratory infection and orange cylinder represents the viral-viral co-infections.)

Of the 471 ILI cases enrolled for laboratory testing, 209 (44.4%) samples tested positive for respiratory viral infection, whereas 262 (55.6%) tested negative for all respiratory viruses (Table 1). Almost equal proportions of male (101/209; 48.3%) and female (108/209; 51.7%) were infected with respiratory viruses. The Median age of ILI patients with respiratory viral infections was 3 years old (IQR: 1–8 years). There was a decline in the percentage of cases that tested positive for respiratory viruses with age. Positive rates for respiratory viruses were higher in age groups under 5 years old (more than 65%) than in age ranges 56 to 65 (2/20) 10%. Figure 2 depicts the positive rates of single infection and viral-viral co-infection.

The prevalence of viral-viral co-infections with respiratory viruses was 3.8% (8/209) across all samples, with children over 16 having a lowest positivity rate than those under 5 years old. Among the positive respiratory virus cases, mixed viral infection was detected with the combination of HCoV-HKU1 and HCoV-NL63 (*N* = 1); IAV (H3N2) and RSV-A (*N* = 1); rhinovirus and HCoV-HKU1 (*N* = 1); IBV and RSV-A (*N* = 3), and hMPV and RSV-A (*N* = 2). The prevalence of viral-viral co-infection among Nicobarese in Car Nicobar is shown in Table 2.

**Table 2:** Detection of viral-viral co-infection cases with ILI during 2021 and 2022

Co-infection	Prevalence (*N* = 8)
HCoV-HKU1 + HCoV-NL63	1
IBV + RSV-A	3
hMPV + RSV-A	2
IAV(H3N2) + RSV-A	1
Rhinovirus + HCoV-HKU1	1

*HCoV* Human Corona virus, *IBV* Influenza B virus, *RSV-A* Respiratory Syncytial Virus, *hMPV* Human metapneumovirus, *IAV* Influenza A virus, *HKU1* Hong Kong University 1, *NL63* Netherlands 63

### Viral etiology

**Fig. 3:**
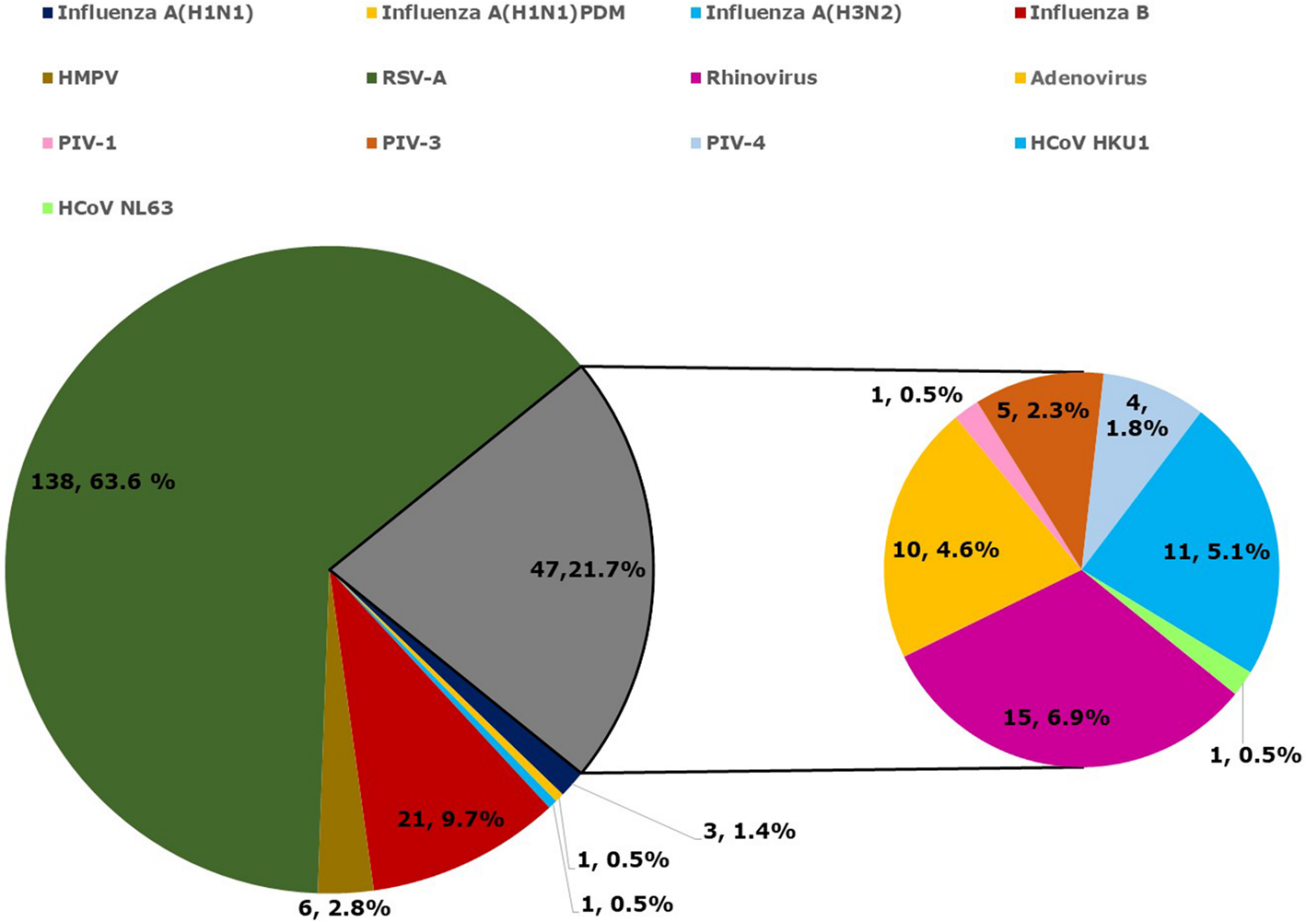
Distribution of respiratory viruses in Car Nicobar Island (Grey section represent the distribution of Rhinovirus, Adenovirus, PIV-1, PIV-3, PIV-4, HCoV-HKU1, HCoV-NL63.)

Among the enrolled cases (*N* = 471), 209 were positive for respiratory viral infections. Of these respiratory viral infections, 201 patients (96.2%) tested positive for a single respiratory viral infection, and 8 (3.8%) tested positive for mixed respiratory viral infection. Among positive respiratory viruses, RSV-A was the most often found respiratory virus, i.e., 63.6%, followed by influenza B virus (9.7%), human rhinovirus (6.9%), HCoV-HKU1 (5.1%), PIV-1 (0.5%), PIV-4 (1.8%), and PIV-3 (2.3%), and hMPV (4.6%). The percentage of HCoV-NL63 and Influenza A viruses were lower than 2.0%. The percentage distribution of respiratory viruses in Car Nicobar was depicted in Fig. 3.

### Age and gender distribution

The distribution of respiratory virus infection by age and gender was determined by evaluating the data. Gender-wise analysis revealed that adenovirus, HCoV, and RSV-A were reported more frequently among females than males. However, influenza B virus, hMPV, and rhinovirus were reported high among males compared to females. Other respiratory viral pathogens did not have any gender predominance. Additionally, there were no age-related changes in respiratory viruses that were statistically significant ()(*p* ≤ 0.05). Influenza A virus, Influenza B virus, RSV-A, HAdV, and PIV were most commonly observed among children under the age of five.

**Fig. 4:**
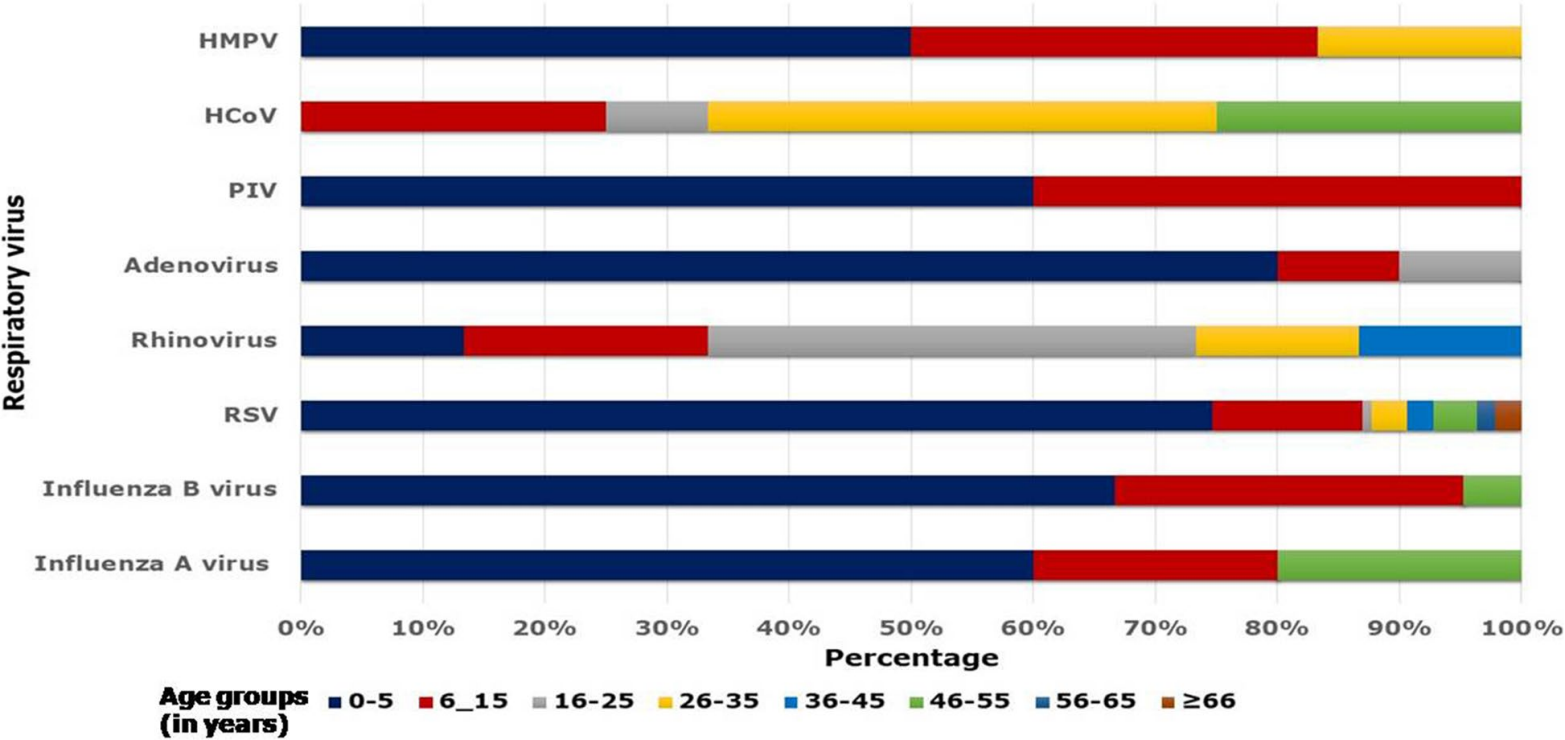
Respiratory viruses among different age groups

Viral pathogens such as hMPV, HCoV, PIV, and influenza virus were found less frequently in children aged 6 to 15 years. While rhinovirus was prevalent in those aged 16 to 25, HCoV was found in those aged 26 to 35. A spectrum of respiratory viruses among different age groups is shown in Fig. 4.

### Respiratory viruses among children

Viral respiratory infections were more common in children less than the age of five and in this study, RSV-A was the most predominantly reported virus among them. Meanwhile, the influenza B virus was prevalent among children. In addition, few cases of other respiratory viruses such as HAdV, hMPV, and PIV were reported.

### Seasonal distribution

**Fig. 5:**
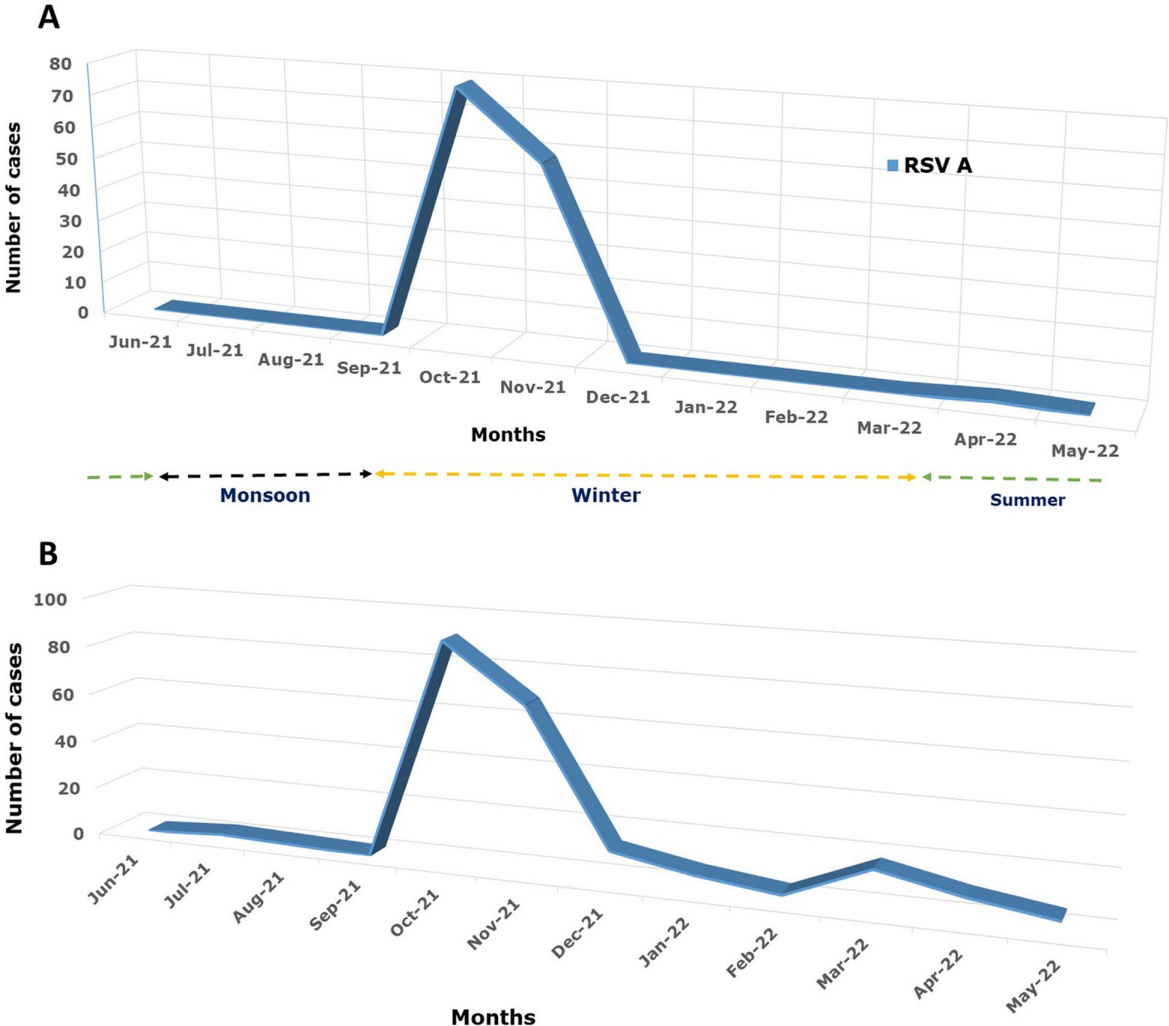
**A** Month wise distribution of RSV-A and **B**. Depicts the month wise distribution of the respiratory viruses

The viral respiratory infection was analyzed as per its seasonal variation. From June 2021 to May 2022 (Fig. 5), RSV-A has a seasonal peak in October 2021. The influenza B virus also increased in October, November, and December 2021. Similarly, hMPV was prevalent during the same seasonal months when RSV-A was on upsurge. HAdV was distributed throughout the year sporadically. However, human rhinovirus and PIV-1, 3, and 4 has increased from the month January 2022 to May 2022. Meanwhile, common coronaviruses HCoV –HKU1 and HCoV-NL63 were also recorded in the months of July 2021, February 2022, March 2022, and April 2022, respectively.

### Association with the weather

**Fig. 6:**
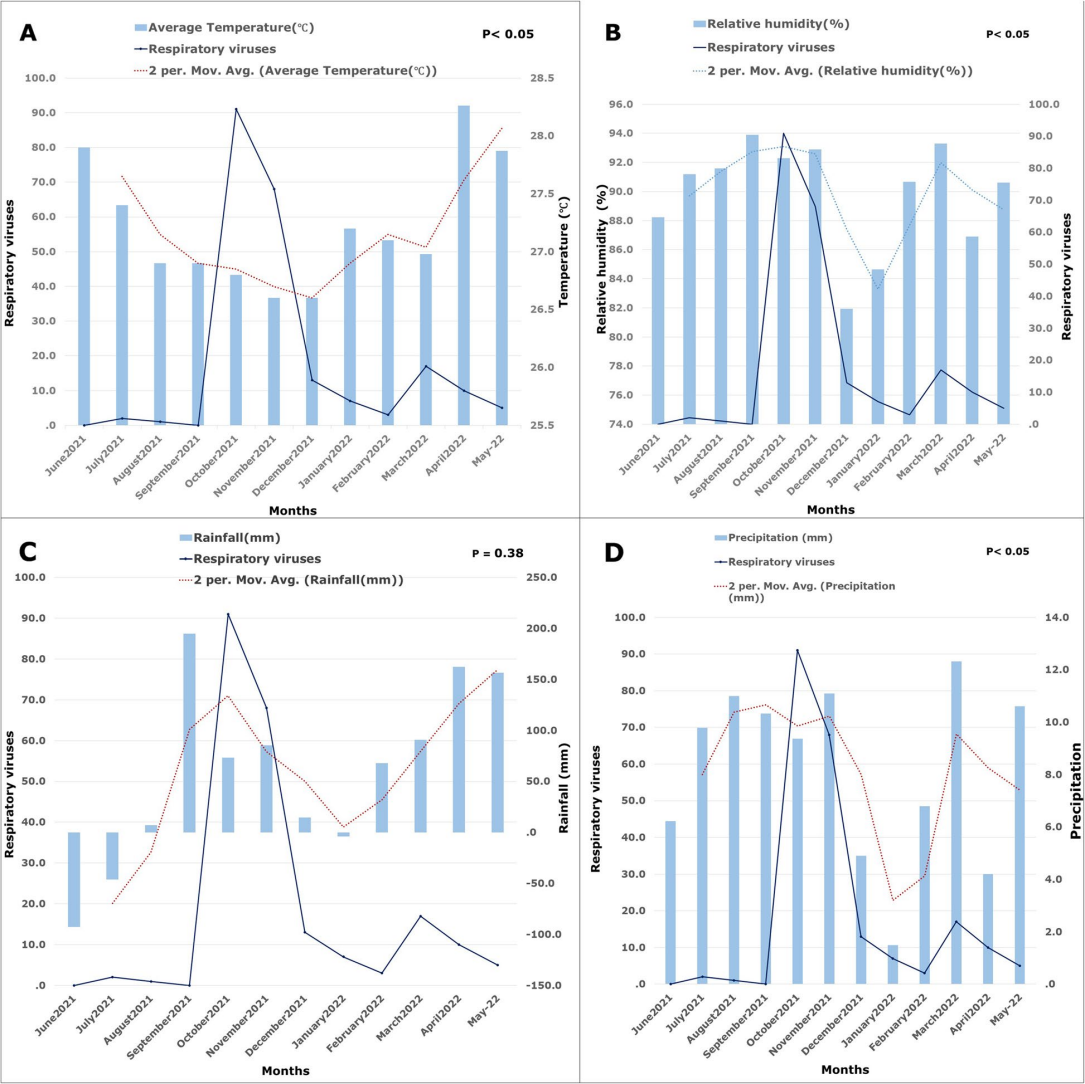
Correlation between the upsurge of respiratory viral infection and Climatic conditions [(**A**) temperature, (**B**) relative humidity, (**C**) rainfall, and (**D**) precipitation]

The annual mean temperature and relative humidity were 27.2 °C and 89.84% respectively. The monthly average (mean) temperature in Car Nicobar Island was low in the month of November 2021 (mean (x̄): 26.6; CI: 26.4 – 26.9) followed by October 2021 (x̄: 26.8; CI: 26.7 – 27.0). Respiratory viral infections were recorded as being low during this period. However, the highest temperature was recorded in the month of April 2022 (x̄: 28.3; CI: 27.9 – 28.6) followed by May 2022 (x̄: 27.9; CI: 27.5 – 28.3) and June 2021(x̄: 27.9; CI: 27.6 – 28.1). There were very few respiratory viruses reported in April (2022) and May (2022), and no cases were reported in June 2021. There was a significant relationship (*p* < 0.05) between the monthly average temperature and the rise in respiratory viruses. In addition, precipitation was recorded as high in March 2022 (x̄: 12.3; CI: 3.6 – 25.8) followed by November 2021 (x̄: 11.1; CI: 7.9 – 14.5). In this period, more frequent respiratory viral infections were reported. There was a significant correlation between monthly average precipitation and a surge of respiratory viruses (*p* < 0.05). Moreover, relative humidity was recorded as high during the month of September (x̄: 93.9; CI: 92.4 – 95.4), October (x̄: 92.3; CI: 90.2 – 94.2), November (x̄: 92.9; CI: 90.8 – 95.0) and March (x̄: 93.3; CI: 87.7 – 98.0). High relative humidity was recorded during the peak of the respiratory viral infection (October and November). There was a significant association between humidity and the respiratory viruses reported (*p* < 0.05). However, rainfall was recorded high in September (x̄: 194.9; CI: 182.5 – 206.5) followed by April (x̄: 162.4; CI: 150.5 – 172.8). Average rainfall was recorded during the upsurge of respiratory viral infections (RSV-A, and influenza B virus). The relative association between rainfall and respiratory viruses was not significant (*p* = 0.38). The prevalence of respiratory viruses increased when there was a moderate temperature, and rainfall. However, relative humidity and precipitation were high during the surge of respiratory viruses (Fig. 6).

## Discussion

For many years, researchers have studied and disputed the factors that underpin the seasonality of respiratory virus infections. Temperature and humidity may affect respiratory virus stability and transmission rates. ARI and SARI is critical public health issues among Nicobarese tribes living in Car Nicobar Island in India. Current research has highlighted the role of environmental variables particularly temperatures and changes in rainfall, relative humidity, and precipitation patterns may have an influence on health of indigenous tribes especially children and elder people in the remote Car Nicobar Island.

Respiratory viruses cause major morbidity and mortality in adults, particularly in elderly with chronic comorbid illnesses [5]. In the Asia–Pacific area, influenza virus circulation varied greatly, with an abnormally high load of influenza B. Overall, influenza B was responsible for 31.4 percent of cases in the Asia–Pacific region [15]. RSV is thought to be the cause of a large number of hospitalizations worldwide, in both industrialized and developing countries. RSV symptoms might range from being asymptomatic to flu-like symptoms to having acute respiratory distress. RSV-A appears to dominate RSV-B in several long-term studies around the world [16]. In this study, the upsurge of RSV-A and influenza B viruses were detected after the COVID-19 pandemic in Car Nicobar Island. Numerous respiratory viruses, including influenza A(H3N2), influenza A(H1N1)PDM, hMPV, HAdV, rhinovirus, and HCoV, were found in this population in a recent report [11].

It is estimated that 3 lakh hospital admissions for RSV-ARI in older adults occur worldwide. Many studies have identified that hospitalization of ARI and SARI as high but the previous studies in Car Nicobar found that patient visited outpatient settings was high [11, 17]. Similarly, in this study, outpatient cases were high compared to hospitalized cases. In the study, there was no discernible gender-specific preponderance in the incidence of ILI cases (*P* > 0.05). Compared to cases that were hospitalized, the proportion of women attending outpatient settings was higher than that of males. There were no cases of COVID-19 during the study, which may be due to the stringent preventive measures that were followed to control the occurrence of SARS-CoV-2 among this indigenous tribe [11].

In this study, the positivity rate of the respiratory viruses was high among children under the age of five. However, there were low positive rates for older age groups (those over 55 years old). Compared to the previous study, respiratory viral infections such as the RSV-A and Influenza B viruses were high among children [11]. Earlier study has reported school going children to be more frequently exposed to influenza virus than other age groups [18]. Among children less than five years, influenza virus, RSV-A, adenovirus, and parainfluenza virus were most commonly identified. This may be due to the schools reopening and the relaxation of stringent measures after the COVID-19 pandemic. The prevalence of respiratory viral pathogens was lower among other age groups. A study from India revealed that the number of respiratory viruses was high among adults, especially those aged 19 to 59 [19].

In most examinations, detection of two or more respiratory viruses at the same time in paediatric patients ranges from 10 to 30% [20, 21]. When compared to respiratory infection alone, previous research indicated that the co-infection of respiratory syncytial virus and human metapneumovirus was protective against the length of hospital stay and hypoxia [21]. In addition, a study revealed that children who had both rhinovirus and RSV infections experienced a shorter duration of hospitalization compared to infants only infected with RSV [22]. Co-infections with the lowest incidence rate have been reported among cases older than 16 years.

Every virus has a seasonal cycle, with peak seasons for activity. A study from the Canary Islands reported that RSV infections showed the same epidemiological pattern as in temperate climates [23]. In a previous study, the majority of viral pathogens exhibited a seasonal pattern of incidence. The prevalence and seasonal variability of respiratory viral agents that are relevant to developing nations must be determined by thorough studies [24]. This island's proximity to the equator and the sea causes its climate to be hot, humid, and consistent, and the islands receive rain from both the northeast and southwest monsoons. The humidity of the Andaman and Nicobar Islands ranges from 76 to 80% on average. Maximum precipitation occurs between May and December [25]. RSV and influenza A virus required a narrow range of humidity, while PIV type 3 needed a dry season. HAdV, influenza A and B, RSV, and hMPV are best suited to cold temperatures [26]. In our study, the prevalence of RSV-A, hMPV, and influenza B virus were reported during the winter season. However, HAdV distribution has been observed all through the year. The rhinovirus has two peaks, one significant in October–November and one moderate in March. During high humidity and precipitation conditions with moderate temperature and rainfall, a surge of respiratory viruses was reported. There was a significant association between respiratory virus infection and climatic factors (*P* < 0.05).

India's Nicobar Islands are a stretch of tropical islands that are situated in the Bay of Bengal, strategically close to Southeast Asian (SEA) nations including Myanmar, Thailand, and Indonesia. Poachers from SEA countries to the Nicobar Islands exploit living marine resources and pose a serious threat of transmitting emerging infectious diseases [27]. Recent studies from Thailand, revealed the predominant influenza viruses, RSV, and adenoviruses among children with SARI and pneumonia [28, 29]. The current study confirmed the presence of influenza viruses and RSV-A among the Nicobarese tribe in Car Nicobar, which is located on the international border line between India and Thailand. This island's pattern of respiratory virus infection differed from that of India's mainland. It may be due to the geographical location and climatic factors of this island. Respiratory viruses like Influenza A virus and SARS-CoV-2 has a potency of transmission from animal to humans or human to animals [30]. Since Car Nicobar Island is a tribal reserve, only people of the Nicobarese tribe are allowed to enter. This population is at risk for emerging and re-emerging viral strains due to reasons such as travel by these people to other regions, hunting activities on these islands, the rearing of pigs, and migrating birds.

Limited treatment options are available for the respiratory virus infections. Some of the antiviral drugs are prescribed for supportive care to reduce the severity of respiratory viral infection. Broad- spectrum antiviral agents like Ribavirin was used for the treatment of RSV,CoV, Adenovirus and Parainfluenza virus. Further, neuraminidase inhibitor such as Oseltamivir was recommended for the treatment of Influenza virus. SARS-CoV-2 causes a wide range of wild and farm animals, humans as well as birds. An experimental study revealed a antiviral activity of Ivermectin against CoV virus.Genomic variants of this virus may cause future epidemic or pandemic due to the rapid evolution of corona viruses [31, 32].

In light of current data, we declare the upsurge of respiratory infections, especially RSV-A and influenza B viruses were predominant among tribal children. Increased activity of RSV-A was seen among children; nevertheless, the transmission of other respiratory viruses like PIVs, rhinovirus, HCoV, and HAdV were all still at low levels. Additionally, a small number of patients had influenza A (H1N1) pdm09 at the same time. The treatment for respiratory viruses has been focused to lowers the influenza symptoms such as fever, and cough. In the future, more investigation needs to be performed to understand the transmission of respiratory viruses in indigenous tribal groups, and the knowledge gathered would aid policymakers in preventing respiratory disease among tribal children in remote islands. This study emphasizes the importance of ongoing respiratory virus surveillance as a public health tool to prevent future epidemics on this remote island. The current investigation will aid in understanding the background activities like age-related factors, seasonal variations, and climatic factors with the pandemic potential to cause respiratory infections in Car Nicobar, the remote islands.

## Limitations

The demographic and clinical data was collected from the patients attending inpatient and outpatient settings of the hospital. Only ILI and SARI patients were recruited while chronic respiratory cases were not included in this study. Due to unwillingness and hesitance of indigenous Nicobarese tribe, the enrolment of participant (sample collected) in this study was low.The study was limited with patients accomplish with the given case definition and eligibility criteria.

## Conclusion

This study looked into the causes of acute respiratory viral infections in Nicobarese tribes in Car Nicobar, India. Upsurges of RSV-A and influenza B virus on this remote island were identified during this study period. The respiratory viruses reported on this island had a significant association with age factors and climatic conditions. Nicobarese children under 5 years were more commonly affected with these respiratory viruses. Further, seasonal pattern of these respiratory viruses in this remote island was revealed through this study. These findings emphasize the need for continuous surveillance of respiratory viral infections and their types in the remote islands where the indigenous people are inhabited. This type of research is necessary for improving and optimizing diagnostic strategies as well as developing approaches for preventing emerging respiratory viral infections.

## Supplementary Information

Supplementary Material 1.

## Data Availability

All data generated or analyzed during this study are included in this published article and its supporting information files.
