# Supplementary material for: Seasonal distribution and upsurge of respiratory viruses among indigenous tribes with ILI and SARI in a far-flung Car Nicobar Island
Source: BMC Infect Dis. 2024 Jun 28;24:651. doi: 10.1186/s12879-024-09536-1 (PMC11212252; doi:10.1186/s12879-024-09536-1)
Supplement: Supplementary file 1 — Supplementary Material 1. [file 12879_2024_9536_MOESM1_ESM.docx]

**CASE PROFORMA**

**Name of CHC / PHC:**

| Date of Onset of Symptoms 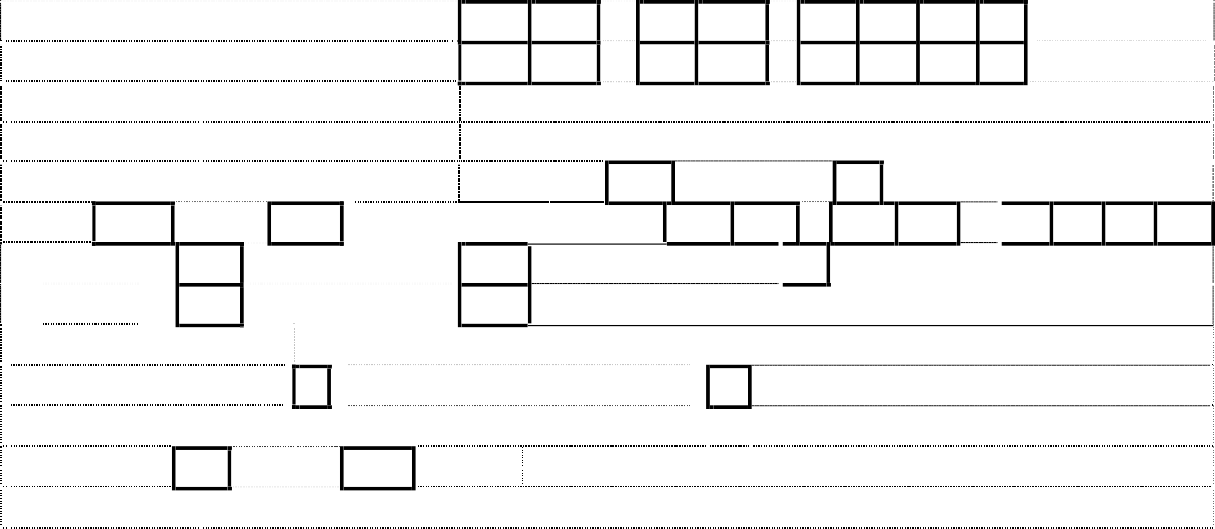 Study ID:  Patient Reg Number: Contact number:  Name of Health Facility: Patient Name:  Age: Year  Specimen: Informant  Month Nasal Swab Self  Gender: Male Date of Birth *D D*  Female  *M M Y Y Y Y*  Throat swab Caregiver  Nasopharyngeal swab  Complete address:  Height (cm): Pregnancy :  Village/Town/City: Rural  Weight (kg):  District:  Urban  Yes  No  if Yes, Gestational age in months:  For children under 5 year: Mid arm circumference (cm): | *D* | *D* | - *M* | *M* | - *Y* | *Y* | *Y Y* |
| --- | --- | --- | --- | --- | --- | --- | --- |
| Date of Sample Collection | *D* | *D* | - *M* | *M* | - *Y* | *Y* | *Y Y* |

| **Exposure History** | **Yes** | **No** |  | **Exposure History** | **Yes** | **No** |
| --- | --- | --- | --- | --- | --- | --- |
| Similar illness in family/neighbor |  |  |  | Smoking (self)/ Smoker in family |  |  |
| Exposure to poultry/dead bird |  |  |  | Exposure to farm animals |  |  |
| No. of family members sleeping in  same room |  | |  | H/o travel abroad in past 14 days  prior to onset |  |  |

| **Symptoms** | **Yes** | **No** |  | **Symptoms** | **Yes** | **No** |
| --- | --- | --- | --- | --- | --- | --- |
| Fever/History of fever (< 7 days) |  |  |  | Chills |  |  |
| Rigors |  |  |  | Cough |  |  |
| Sore throat |  |  |  | Haemoptysis |  |  |
| Ear ache/ discharge |  |  |  | Nasal Discharge/stuffiness |  |  |
| Body-ache |  |  |  | Headache |  |  |
| Chest Pain |  |  |  | Malaise/Fatigue |  |  |
| Vomiting/nausea |  |  |  | Abdominal pain |  |  |
| Breathlessness/ difficulty breathing |  |  |  | Diarrhea |  |  |
| Seizures |  |  |  |  | | |
| Other Symptoms: | | | | | | |

|  | |
| --- | --- |
| **Signature** |  |
| **Name of interviewer** |  |
